# Proteomic Analysis of GLUT4 Storage Vesicles Reveals Tumor Suppressor Candidate 5 (TUSC5) as a Novel Regulator of Insulin Action in Adipocytes*[Fn FN2]

**DOI:** 10.1074/jbc.M115.657361

**Published:** 2015-08-03

**Authors:** Daniel J. Fazakerley, Sheyda Naghiloo, Rima Chaudhuri, Françoise Koumanov, James G. Burchfield, Kristen C. Thomas, James R. Krycer, Matthew J. Prior, Ben L. Parker, Beverley A. Murrow, Jacqueline Stöckli, Christopher C. Meoli, Geoffrey D. Holman, David E. James

**Affiliations:** From the ‡ Charles Perkins Centre, School of Molecular Bioscience, and; ‖ School of Medicine, University of Sydney, Sydney, New South Wales 2006, Australia,; § The Garvan Institute of Medical Research, Sydney, New South Wales 2010, Australia, and; the ¶ Department of Biology and Biochemistry, University of Bath, Bath BA2 7AY, United Kingdom

**Keywords:** adipocyte, glucose transporter type 4 (GLUT4), insulin, insulin resistance, membrane trafficking, tumor suppressor candidate 5 (TUSC5)

## Abstract

Insulin signaling augments glucose transport by regulating glucose transporter 4 (GLUT4) trafficking from specialized intracellular compartments, termed GLUT4 storage vesicles (GSVs), to the plasma membrane. Proteomic analysis of GSVs by mass spectrometry revealed enrichment of 59 proteins in these vesicles. We measured reduced abundance of 23 of these proteins following insulin stimulation and assigned these as high confidence GSV proteins. These included established GSV proteins such as GLUT4 and insulin-responsive aminopeptidase, as well as six proteins not previously reported to be localized to GSVs. Tumor suppressor candidate 5 (TUSC5) was shown to be a novel GSV protein that underwent a 3.7-fold increase in abundance at the plasma membrane in response to insulin. siRNA-mediated knockdown of TUSC5 decreased insulin-stimulated glucose uptake, although overexpression of TUSC5 had the opposite effect, implicating TUSC5 as a positive regulator of insulin-stimulated glucose transport in adipocytes. Incubation of adipocytes with TNFα caused insulin resistance and a concomitant reduction in TUSC5. Consistent with previous studies, peroxisome proliferator-activated receptor (PPAR) γ agonism reversed TNFα-induced insulin resistance. TUSC5 expression was necessary but insufficient for PPARγ-mediated reversal of insulin resistance. These findings functionally link TUSC5 to GLUT4 trafficking, insulin action, insulin resistance, and PPARγ action in the adipocyte. Further studies are required to establish the exact role of TUSC5 in adipocytes.

## Teaser

**Background:** We searched for novel regulators of insulin-stimulated glucose transport in adipocytes.

**Results:** Tumor suppressor candidate 5 (TUSC5) colocalized with GLUT4, and manipulation of TUSC5 expression levels affected insulin-regulated glucose transport.

**Conclusion:** TUSC5 is a novel regulator of insulin-stimulated glucose transport.

**Significance:** TUSC5 contributes to insulin-sensitizing effects of PPARγ agonists in adipocytes.

## Introduction

Insulin regulates blood glucose levels in part by stimulating the translocation of GLUT4 from an intracellular compartment to the plasma membrane (PM)^6^ of adipocytes and myocytes (1). This process is defective in insulin resistance (2).

In the absence of insulin, GLUT4 resides within specialized insulin-sensitive 50–70-nm storage vesicles (3, 4), termed GLUT4-storage vesicles (GSVs). These vesicles are distinct from the general endosomal recycling pathway, effectively sequestering GLUT4 and maintaining low levels of PM GLUT4 in the absence of insulin. Insulin signals via the canonical phosphatidylinositol 3-kinase (PI3K)/AKT signaling pathway to trigger the release, translocation, docking, and fusion of GSVs with the PM (5, 6). Mobilization of GSVs to the PM upon insulin stimulation permits a large augmentation of cell surface GLUT4 levels and thus glucose transport into the cell. Activation of AKT is necessary and sufficient for GSV exocytosis (5), and this is thought to be primarily mediated through AKT-dependent phosphorylation and inactivation of the TBC1D4/AKT substrate of 160 kDa (AS160), a RAB-GAP residing on GSVs (5, 7–9). The identification of TBC1D4 as an AKT substrate and GSV-associated protein represents a major advance in our understanding of the intersection between insulin signaling and GLUT4 traffic. In addition to TBC1D4, established GSV resident proteins have been reported to regulate GLUT4 traffic in different ways as follows: those proteins that regulate GSV formation (*e.g.* sortilin (10)); those that regulate GSV trafficking either as part of the signaling response or by directing membrane traffic (*e.g.* TBC1D4 (9), RAB10 (11–13), and VAMP2 (14)); and those that are delivered to the PM as cargo within GSVs (*e.g.* GLUT4, insulin-responsive aminopeptidase (IRAP) (15), and low density lipoprotein receptor-related protein 1 (LRP1) (16)).

Insulin-regulated GLUT4 trafficking in adipocytes is defective in insulin-resistant conditions. PPARγ agonists, such as the thiazolidinediones (*e.g.* rosiglitazone), enhance whole body insulin sensitivity (17–19) and restore insulin-regulated glucose transport in insulin-resistant adipocytes (20–24). Furthermore, PPARγ activation in adipose tissue alone has been reported to be sufficient for whole body insulin sensitization (23). It has been proposed that the insulin sensitizing activity of PPARγ activation may be mediated via a range of pathways, including the following: enhanced adipocyte differentiation (25, 26); altered expression of key intermediates such as GLUT4 (20, 24, 27–29), IRS1, IRS2, PI3K P85 (23), and PTEN (30); reduced production of inflammatory cytokines (31); altered GLUT4 recycling (22); reduced oxidative stress (21); increased mitochondrial biogenesis (32); and inhibition of mitochondrial respiration (33). Interestingly, no individual PPARγ target genes, other than *GLUT4* itself, have been found to participate directly in GSV trafficking.

Given the importance of the GSV compartment to the insulin response, and recent advances in the sensitivity of mass spectrometry instruments, we sought to expand the repertoire of known GSV proteins and to identify novel regulators of GLUT4 traffic by targeting GSVs for proteomic analysis (9, 16). Our analysis revealed a number of novel GSV proteins, which likely denote novel regulatory features of GSVs. One such protein, tumor suppressor candidate 5 (TUSC5), was rapidly mobilized to the cell surface following insulin stimulation. Genetic manipulation of *Tusc5* expression placed TUSC5 as a positive regulator of insulin-stimulated glucose transport. Furthermore, we provide evidence that enhanced TUSC5 expression forms part of the insulin-sensitizing action of the PPARγ agonist rosiglitazone. Our findings implicate TUSC5 as a novel regulator of insulin action in adipocytes.

## Experimental Procedures

### 

#### 

##### Immunoisolation of GSVs from Primary Rat Adipocytes

Adipose cells from epididymal fat pads of male Wistar rats weighing 180–200 g were prepared by collagenase digestion as described previously (34). Adipocytes at 40% cytocrit were stimulated with 20 nm insulin for 20 min at 37 °C. The cells were homogenized in HES buffer (20 mm HEPES, pH 7.0, 0.5 mm EGTA, 250 mm sucrose) supplemented with protease inhibitors and processed as described previously (35) to obtain a post-high density microsome supernatant (pHS) or low density microsomes (LDM). GLUT4 vesicles were purified by immunoisolation using an anti-GLUT4 antibody (36) prebound to the maltose-binding protein-protein A construct attached to amylose resin (New England Biolabs) as described previously (37). After a 2-h incubation, the column was washed with HES buffer, and the GLUT4 vesicles were eluted in 40 mm maltose, 100 mm Tris-HCl, pH 7.4, and protease inhibitors. The eluted fractions were concentrated with Amicon ultra spin filters (Millipore 10,000 molecular weight cutoff) to reduce the elution volume, and SDS was added to 2% w/v final concentration.

##### Immunoisolation of GSVs from 3T3-L1 Adipocytes

3T3-L1 adipocytes were serum-starved in basal DMEM (DMEM,GlutaMAX, and 0.2% bovine serum albumin (BSA)) for 2 h prior to 100 nm insulin stimulation for 20 min where indicated. Cells were washed with ice-cold PBS and harvested in ice-cold HES-I buffer (20 mm HEPES, pH 7.4, 1 mm EDTA, 250 mm sucrose containing Complete protease inhibitor mixture (Roche Applied Science)). All subsequent steps were carried out at 4 °C. Cells were homogenized by passing through 22- and 27-gauge needles prior to centrifugation at 500 × *g* for 10 min. The supernatant was centrifuged at 13,550 × *g* for 12 min to yield a pellet (PM/mitochondria/nuclei) and a supernatant fraction consisting of cytosol, LDM, and high density microsomes (HDM). The supernatant was then centrifuged at 21,170 × *g* for 17 min to obtain the HDM pellet and the supernatant containing the LDM and cytosol fractions (herein referred to as the post-HDM supernatant, pHS). Protein concentration of pHS was determined using BCA assay (Thermo Scientific). Equal amounts of protein from each pHS was incubated with protein G-agarose beads (GE Healthcare) conjugated either to anti-GLUT4 (1F8 monoclonal anti-mouse antibody) or IgG control antibody (Sigma) for 2 h with rotation. Beads were washed three times with HES-I, followed by two washes with PBS, and precipitated proteins were eluted in 2× SDS-PAGE loading buffer (65.8 mm Tris-HCl, pH 6.8, 2.1% SDS, 26.3% (v/v) glycerol, 0.01% bromphenol blue) containing 20 mm DTT.

##### Protein Fractionation by SDS-PAGE for Mass Spectrometry

Immunoisolated proteins were reduced with 10 mm DTT for 1 h at room temperature and alkylated with 25 mm iodoacetamide for 30 min in the dark before iodoacetamide was quenched with 10 mm DTT. Samples were diluted in NuPAGE LDS sample buffer (141 mm Tris base, 2% lithium dodecyl sulfate, 10% glycerol, 0.51 mm EDTA, 0.22 mm SERVA Blue G, 0.175 mm phenol red) (Life Technologies, Inc.) prior to SDS-PAGE. Samples were run on an 8–12% bis/acrylamide SDS-PAGE Precast NuPAGE gel (Life Technologies, Inc.). Gels were stained with Sypro Ruby protein gel stain (Life Technologies, Inc.) according to the manufacturer's instructions and imaged on a Fujifilm FLA-5100 (Fujifilm).

##### In-gel Digestion

Each lane was cut into 10 separate fractions for in-gel digestion. Gel fractions were washed in 50% acetonitrile (MeCN, Thermo Scientific) in 50 mm triethylammonium bicarbonate (TEAB, Sigma) at room temperature for 15 min, vortexing occasionally. Gel pieces were dehydrated in 100% MeCN at room temperature for 5 min and rehydrated in 50 mm TEAB at room temperature for 5 min. This dehydration/rehydration procedure was repeated but with the rehydration buffer containing 10 ng/μl trypsin (Promega) in 50 mm TEAB. Digest was carried out overnight at 37 °C. Peptides were solubilized by the addition of formic acid (Thermo Scientific) to 5% and incubation at 37 °C for 30 min. To extract peptides, gel pieces were dehydrated using 100% MeCN and incubated at 37 °C for 15 min. Peptides were transferred to a new tube and dried and then resuspended and acidified in 1% v/v trifluoroacetic acid (TFA, Thermo Scientific). Resuspended peptides were centrifuged for at 21,000 × *g* for 10 min at 4 °C. Each sample was desalted using C18 membranes (3 m) as described previously (38).

##### Nano-reverse Phase Liquid Chromatography-Electrospray Ionization-Tandem Mass Spectrometry

Peptides were resuspended in 0.5% acetic acid and separated by reverse phase liquid chromatography on an in-house packed 17-cm × 75-μm Reprosil-Pur C18-AQ column (1.9 μm, Dr. Maisch) using an EASY nLC-II nanoHPLC (Proxeon). The HPLC gradient was 0–40% solvent B (solvent A, 0.5% acetic acid; solvent B, 90% acetonitrile, 0.5% acetic acid) over 90 min at a flow of 250 nl/min. MS was performed using an LTQ-Orbitrap Velos Pro (Thermo Scientific). An MS scan (300–1750 *m*/*z*; MS AGC 3 × 10^6^) was recorded in the Orbitrap set at a resolution of 60,000 at 400 *m*/*z* followed by data-dependent collision-induced dissociation MS/MS of the 20 most intense precursor ions. An MS scan (300–1750 *m*/*z*; automatic gain control 3 × 10^6^) was recorded in the Orbitrap set at a resolution of 60,000 at 400 *m*/*z* followed by data-dependent collision-induced dissociation MS/MS of the 20 most intense precursor ions. Parameters for collision-induced dissociation were as follows: normalized energy 35, dynamic duration 60 s, maximum injection time 150 ms, and tandem MS automatic gain control 4 × 10^5^.

##### Mass Spectrometric Data Analysis

Raw mass spectrometry data were processed using the MaxQuant software (39) version 1.2.3.3 using the default settings with minor changes as follows: oxidized methionine (Met), acetylation (N-terminal protein) were selected as variable modifications and carbamidomethyl as fixed modification. A maximum of two missed cleavages was permitted, 10 peaks per 100 Da, MS/MS tolerance of 20 ppm, and a minimum peptide length of 7. The “matching between runs” algorithm was enabled (time window = 2 min) to transfer identifications between adjacent fractions. Database searching was performed using the Andromeda search engine integrated into the MaxQuant environment (40) against the rat Uniprot database (October, 2013). Protein, peptide, and site false discovery rate thresholds in MaxQuant were each set to a maximum of 1%. Relative protein abundances were estimated using the “Label-free Quantification Method” (LFQ) algorithm (41) within the MaxQuant environment. Resulting “LFQ” intensities were used for analysis.

Analyses of mass spectrometry data were performed in R programming environment (42). All data were log2-transformed and normalized using the median absolute deviation (43) method. Missing values in IgG control and insulin-stimulated samples of each experiment were imputed 20 times and averaged. Imputation was performed by generating a Gaussian distribution with mean (μ) set to the minimum value for each condition, and the standard deviation (σ) was the σ of the 1st quartile of the distribution for each condition. Normally distributed random variables from this minimum value distribution were used to replace the missing values.

##### Statistical Analysis

Only proteins identified in all replicates (*n* = 2 for LDM and *n* = 2 for pHS) were retained for statistical analysis. The top 5% most variable data (determined by standard deviation) were removed prior to analysis. We filtered for high confidence GSV-resident proteins by selecting proteins with features similar to a positive control set (GLUT4, IRAP, VAMP2, and sortilin). These features were enrichment of proteins within GSVs and LFQ intensity as a measure of abundance and insulin responsiveness. For analysis of both enrichment (basal *versus* IgG) and insulin responsiveness (Insulin *versus* basal), the significance of each protein was calculated using a moderated *t* test from LIMMA package (44). Test statistics (*t* statistics) were obtained separately for each immunoisolation method (LDM or pHS) and converted to *Z*-scores. An integrated *p* value was derived by testing for proteins that were altered in both LDM and pHS in the positive quadrant for enriched proteins using directPA package (45). Only proteins with LFQ intensities greater than the population median were considered for further analysis. An integrated *p* value was also derived for the insulin responsiveness of proteins using the same method, by testing for proteins altered in both LDM and pHS in the negative quadrant.

Nonlinear regressions to calculate ED_50_ values, linear regressions to determine *r*^2^ values, *t* tests, and ANOVAs were performed using GraphPad Prism version 6.00 for Windows (GraphPad software). Specific statistical tests are specified in figure legends.

##### Cloning of TUSC5

The *Tusc5* gene was cloned from 3T3-L1 cDNA into pcDNA3.1 and subsequently into pBABE-puro, using Gibson assembly cloning (46). The sequences of cloning primers are available upon request. Constructs were confirmed by sequencing.

##### Cell Culture

3T3-L1 fibroblasts were passaged at ∼60% confluency in Dulbecco's modified Eagle's medium supplemented with 10% fetal bovine serum (FBS), GlutaMAX (Life Technologies, Inc.) at 37 °C with 10% CO_2_. Differentiation was induced at 100% confluence by addition of 350 nm insulin, 0.5 mm 3-isobutyl-1-methylxanthine, 250 nm dexamethasone, and 400 nm biotin for 3 days. Cells were then incubated in the presence of 350 nm insulin for 3 days. Adipocytes were used between days 10 and 12 after initiation of differentiation. For overexpression of TUSC5 and/or hemagglutinin (HA)-tagged-GLUT4 3T3-L1, fibroblasts were infected with pBABE puro retrovirus (empty vector control) and pBabepuro-TUSC5 retrovirus alone or in combination with pWZLneo HA-GLUT4 retrovirus. Puromycin (2 μg/ml) was used for selection of cells infected with pBABE puro retrovirus. Cells were also selected with geneticin (800 μg/ml) if infected with pWZLneo HA-GLUT4 retrovirus. For induction of insulin resistance by TNFα, adipocytes were incubated with 2 ng/ml TNFα for 96 h. Rosiglitazone (10 μm) treatments were carried out during the final 48 h of TNFα treatment.

##### Subcellular Fractionation

3T3-L1 adipocytes were serum-starved in basal DMEM for 2 h prior to stimulation with 100 nm insulin for 20 min before being subjected to subcellular fractionation. Cells were washed with ice-cold PBS and harvested in ice-cold HES-I buffer. All subsequent steps were carried out at 4 °C. Cells were homogenized by passing through 22- and 27-gauge needles prior to centrifugation at 500 × *g* for 10 min. The supernatant was centrifuged at 13,550 × *g* for 12 min to pellet the PM and mitochondria/nuclei, and the supernatant fraction consisted of cytosol, LDM, and HDM. The supernatant was then centrifuged at 21,170 × *g* for 17 min to pellet the HDM fraction. The supernatant was again centrifuged at 235,200 × *g* for 75 min to obtain the cytosol fraction (supernatant) and the LDM fraction (pellet). The PM and mitochondria/nuclei pellet were resuspended in HES-I and centrifuged at 13,550 × *g* for 12 min. The PM and mitochondria/nuclei pellet were resuspended in HES-I and layered over a high sucrose HES-I buffer (1.12 m sucrose, 0.05 mm EDTA, 10 mm HEPES, pH 7.4) and centrifuged at 111,160 × *g* for 60 min in a swing-out rotor. The PM fraction was collected above the sucrose layer, and the pellet was the mitochondria/nuclei fraction. All the fractions were resuspended in HES-I. Protein concentration for each fraction was performed using BCA assay (Thermo Scientific).

##### Mouse Tissue Lysate Production

Tissues were isolated from the mouse and homogenized in HES-SDS (2% (w/v) SDS) with a Dounce homogenizer followed by sonication. Samples were centrifuged at 13,000 × *g* for 10 min at room temperature. The supernatant was collected, and protein concentration was determined by BCA assay (Thermo Scientific).

##### Immunofluorescence Confocal Microscopy

3T3-L1 adipocytes were seeded onto Matrigel matrix-coated 8-well microslides (Ibidi). Cells were serum-starved in basal DMEM at 37 °C before stimulation with 100 nm insulin for 20 min. Cells were washed in ice-cold PBS, fixed with 3.8% paraformaldehyde, and quenched with 50 mm glycine. Cells were then blocked and permeabilized in 2% BSA and 0.1% saponin in PBS and incubated with anti-GLUT4 (1F8, mouse), anti-TUSC5 (rabbit, Santa Cruz Biotechnology), and anti-EEA1 (Dr. Marvin Fritzler, University of Calgary). Primary antibodies were detected with anti-mouse Alexa-488-conjugated secondary, anti-rabbit Alexa-555-conjugated secondary antibody, and anti-human Alexa-647-conjugated secondary antibody. 4,6′-Diamidino-2-phenylindole, dihydrochloride (DAPI), was used to visualize nuclei (Life Technologies, Inc.). The imaging medium was 2.5% 1,4-diazabicyclo[2.2.2]octane, 5% glycerol in PBS. Optical sections were obtained using the Leica TCS SP8 confocal laser scanning microscope using a HC PL APO 63×/1.20 W CORR CS2 objective and HyD detectors at 22 °C. Images were acquired using Leica LCS software. All images were processed using Fiji (47).

##### Total Internal Reflection Fluorescence (TIRF) Microscopy

3T3-L1 adipocytes or 3T3-L1 adipocytes expressing HA-GLUT4 were seeded onto Matrigel matrix-coated 8-well microslides (Ibidi), serum-starved, and treated with insulin as above. Cells were washed in ice-cold PBS, fixed with 3.8% paraformaldehyde, and quenched with 50 mm glycine. For assessment of only plasma membrane HA-GLUT4 and TUSC5, cells were blocked in 2% BSA in PBS and incubated with anti-HA (Covance, mouse) and anti-TUSC5 (rabbit, Santa Cruz Biotechnology). Primary antibodies were detected with anti-mouse Alexa-488-conjugated secondary and anti-rabbit Alexa-555-conjugated secondary antibodies. For visualization of endogenous GLUT4 and TUSC5 by TIRF, cells were blocked and permeabilized in 2% BSA and 0.1% saponin in PBS and incubated with anti-GLUT4 (1F8, mouse) and anti-TUSC5 (rabbit, Santa Cruz Biotechnology). Primary antibodies were detected with anti-mouse Alexa-488-conjugated secondary antibody and anti-rabbit Alexa-555-conjugated secondary antibody. DAPI was used to visualize nuclei (Life Technologies, Inc.). The imaging medium was 2.5% 1,4-diazabicyclo[2.2.2]octane, 5% glycerol in PBS. Images were acquired on a Nikon Ti-E microscope at 25 °C. Total internal reflection fluorescence microscopy was performed using 488- and 555-nm lasers introduced into the excitation light path through the LApps H-TIRF module (Nikon) angled to image ∼110 nm into cells. Fluorescence was detected using an Andor iXon3 888 EMCCD camera. Data analysis was performed using a combination of Fiji (47), ilastik (48), and Cell Profiler (49).

##### siRNA-mediated Tusc5 Knockdown

7 days post-differentiation, adipocytes were trypsinized (5× trypsin, EDTA) (Life Technologies, Inc.) at 37 °C, washed twice with PBS, and resuspended in electroporation solution (20 mm HEPES, 135 mm KCl, 2 mm MgCl_2_, 0.5% Ficoll 400, 1% DMSO, 2 mm ATP, and 5 mm glutathione, pH 7.6) with 200 nm scrambled (sense 5′-CAGTCGCGTTTGCGACTGGTT-3′) or pooled anti-TUSC5 siRNA (sense 5′-GGAGAACAAGGAUGACCAATT-3′, 5′-GGGCAUCGUUAUCAUCAUUTT-3′, 5′-UCCUCCGUCAUCACCACAUTT-3′). (Shanghai Genepharma). Cells were electroporated at 200 mV for 20 ms using an ECM 830 square wave electroporation system (BTX Molecular Delivery Systems) and seeded onto Matrigel (Corning)-coated coverslips or plates. Cells were assayed 96 h following electroporation.

##### Cell Lysis

3T3-L1 adipocytes were serum-starved in basal DMEM for 2 h before incubation with 0.5 or 100 nm insulin for 20 min where indicated. Cells were washed twice with PBS and lysed 1% SDS in PBS-containing protease inhibitors (Roche Applied Science), 1 mm sodium pyrophosphate, 2 mm sodium orthovanadate, 10 mm sodium fluoride. Cell lysates were sonicated for 12 s and centrifuged at 13,000 × *g*. The protein concentration of the supernatant was determined by BCA assay (Thermo Scientific).

##### SDS-PAGE and Western Blotting

10 μg of protein were resolved by SDS-PAGE and transferred to PVDF membranes. Membranes were blocked in 5% BSA or skim milk powder in TBS-T (0.1% v/v Tween 20 in Tris-buffered saline) for 1 h followed by an overnight incubation at 4 °C with specific primary antibody solutions. Membranes were incubated with an appropriate secondary antibody for 1 h before signals were detected using ECL (Thermo Scientific or Millipore) on the Chemidoc MP (Bio-Rad). Data analysis was performed using ImageJ or Image Lab version 5.2.1 (Bio-Rab). Antibodies detecting AKT, Ser(P)-473 AKT, Thr(P)-308 AKT, TBC1D4, Thr(P)-642 TBC1D4, and Tyr(P)-100 were obtained from Cell Signaling Technology. IRS-1, IRβ, TUSC5, and 14-3-3 were detected using antibodies obtained from Santa Cruz Biotechnology. Antibodies against IRAP and GLUT4 were generated in-house. Densitometry analysis was performed using Image Lab 5.2.1 (Bio-Rad). For determination of TUSC5 level, all molecular weight bands present were included for quantification.

##### qPCR

Total RNA was extracted from cells using TRIzol reagent (Invitrogen). cDNA synthesis was carried out using PrimeScript first strand cDNA synthesis kit (Clontech and Takara Bio Company). PCRs were performed on the LightCycler 480 II (Roche Applied Science) using FastStart Universal SYBR Green Master (Roche Applied Science). Cyclophilin *b* was used asan internal control. The primer sets used were as follows: m*Tusc5*-F, ggtccttgccattgcctctt, and m*Tusc5*-R, tgctgcacactacttcgagac; m*Glut4*-F, gacggacactccatctgttg, and m*Glut4*-R, gccacgatggagacatagc; m*CypB*-F, and ttcttcataaccacagtcaagacc; m*CypB*-R, accttccgtaccacatccat.

##### 2-[^3^H]Deoxyglucose Uptake Assay

3T3-L1 adipocytes were serum-starved in basal DMEM for 2 h before being washed three times in warm PBS and incubated in Krebs-Ringer buffer (KRP) (0.6 mm Na_2_HPO_4_, 0.4 mm NaH_2_PO_4_, 120 mm NaCl, 6 mm KCl, 1 mm CaCl_2_, 1.2 mm MgSO_4_ and 12.5 mm HEPES, pH 7.4) with 0.2% BSA. Cells were stimulated with 0.5 or 100 nm insulin for 20 min. To determine nonspecific glucose uptake, 25 μm cytochalasin B was added to the wells before addition of 2-[^3^H]deoxyglucose (2DOG). During the final 5 min of insulin stimulation, 2DOG uptake was initiated by addition of 2DOG (0.125 μCi/well, 50 μm unlabeled 2DOG) (PerkinElmer Life Sciences). Following three rapid washes in ice-cold PBS, cells were solubilized in 1% (w/v) Triton X-100 in PBS on a shaker for 1 h and assessed for radioactivity by scintillation counting using a β-scintillation counter. All data were normalized to protein content.

##### HA-GLUT4 Assay

Measurement of cell surface HA-GLUT4 as a percentage of total cellular HA-GLUT4 was performed in 96-well plates as described previously (50). Briefly, cells were serum-starved for 2 h in basal DMEM. Cells were stimulated with 0.5 or 100 nm insulin as indicated. Cells were subsequently fixed but not permeabilized, and the amount of HA-GLUT4 present at the plasma membrane was determined from the accessibility of the HA epitope to anti-HA antibody (Covance). Cells were incubated with 20 μg/ml goat anti-mouse Alexa-488-conjugated secondary antibody (Life Technologies, Inc.). Determination of total HA-GLUT4 level was performed in a separate set of wells that underwent the same labeling procedure except that anti-HA staining was performed after permeabilization with 0.1% (w/v) saponin. Total HA-GLUT4 levels were measured separately for each experimental treatment group. Fluorescence (excitation 485 nm/emission 520 nm) was measured using a fluorescent microtiter plate reader (FLUOstar Galaxy, BMG LABTECH). Surface levels of HA-GLUT4 were expressed as a percentage of total HA-GLUT4.

## Results

### 

#### 

##### Proteomic Analysis of GLUT4 Storage Vesicles

**FIGURE 1.**
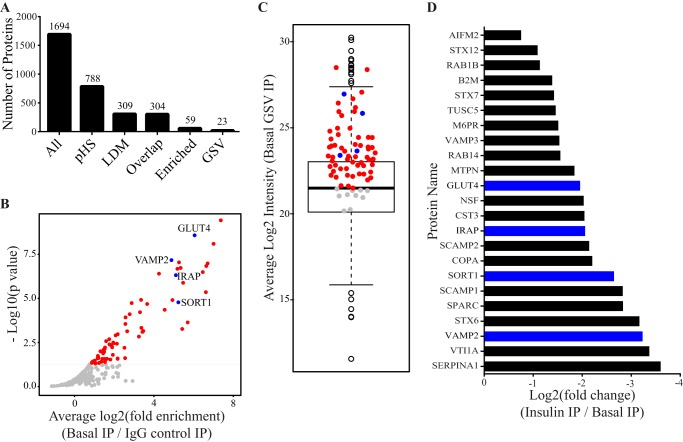
**Proteomic analysis of GLUT4 storage vesicles.** *A*, number of protein groups carried through each filtering step of the analytical workflow (*All*, all proteins identified by mass spectrometry with known contaminants removed; *pHS*, proteins present in GSVs isolated from the pHS starting material (*n* = 2); *LDM,* proteins present in GSVs isolated from the LDM starting material (*n* = 2); *Overlap,* proteins identified in both LDM and pHS (*n* = 4); *Enriched,* proteins enriched within GSVs from unstimulated adipocytes with an average intensity greater than the median (*n* = 4, integrated *p* value < 0.05); *GSV,* enriched proteins that were also insulin-responsive (*n* = 4, integrated *p* < 0.05). *B*, volcano plot depicting proteins enriched within GSVs (*red* and *blue points* are proteins significantly enriched (*n* = 4, integrated *p* < 0.05)). *Blue points* represent known GSV proteins. *C,* boxplot of the average log2 LFQ intensities for all proteins present in GSVs from unstimulated adipocytes. *Blue points* represent known GSV proteins; *red points* represent enriched proteins (*B*) with average log2 LFQ greater than the population median; *gray points* are proteins below the median cutoff. *D*, fold response of 23 proteins that were enriched in GSVs and whose abundance decreased significantly following insulin treatment (*n* = 4, integrated *p* < 0.05). *Blue bars* highlight known GSV proteins. *IP*, immunoprecipitation.

To identify potential regulators of insulin-stimulated GLUT4 trafficking in adipocytes, we performed proteomic profiling of GSVs. We immunoisolated GSVs from both unstimulated and insulin-stimulated primary rat adipocytes using an antibody raised against the C terminus of GLUT4. We used two different membrane preparations derived from differential centrifugation as starting material. One was a mixture of cytosol and small vesicular membranes referred to as pHS. The second was composed of small vesicular membranes (LDM). Immunoisolated vesicles were fractionated by SDS-PAGE prior to peptide extraction for analysis by liquid chromatography-tandem MS (LC-MS/MS). An advantage of this in-gel approach was that it facilitated partial separation of immunoglobulin heavy and light chains from GSV proteins. MS analysis identified 1,694 proteins across all test and control conditions (supplemental Table S1). We identified 309 and 788 proteins in both replicates of the LDM and pHS preparations, respectively (Fig. 1*A*). There was a high degree of overlap in proteins identified using each of the above isolation methods (304/309) (Fig. 1*A*) so both protein lists were merged prior to statistical analysis (304 proteins, *n* = 4).

**FIGURE 2.**
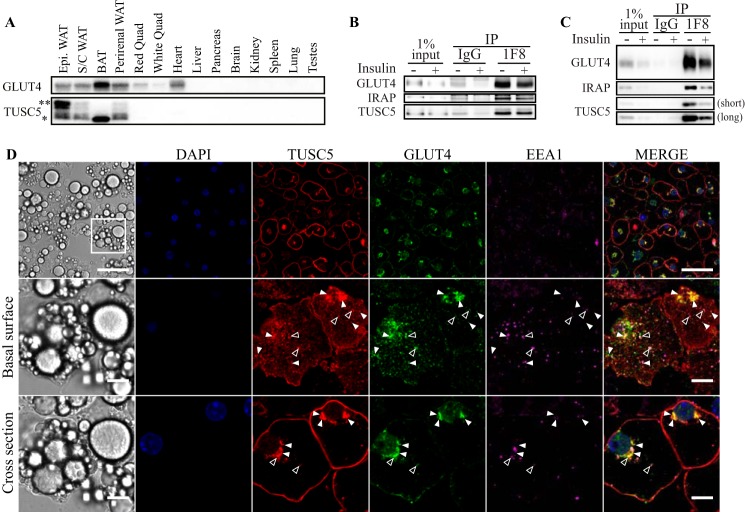
**TUSC5 is a novel GSV protein.** *A*, TUSC5 and GLUT4 expression was assessed in a panel of mouse tissues by Western blotting (representative image of *n* = 2). *Asterisks* denote TUSC5-specific bands of different molecular mass (* = ∼20 kDa, ** = ∼30 kDa). *B*, GSVs were immunoisolated from primary rat adipocytes treated with and without insulin. Abundance of GLUT4 and TUSC5 within the LDM fraction obtained from the pHS starting material and precipitates was determined by immunoblotting (representative of *n* = 3). *C*, GSVs were immunoisolated from 3T3-L1 adipocytes treated with and without insulin. GLUT4, IRAP, and TUSC5 levels were determined by immunoblotting (representative of *n* = 3). Two exposure levels are presented for TUSC5 to permit visualization of TUSC5 in the starting material. *D*, 3T3-L1 adipocytes were serum-starved for 2 h prior to fixation and processing for immunofluorescent imaging by confocal microscopy. Cells were stained for nuclei (DAPI, *blue*), TUSC5 (*red*), GLUT4 (*green*), and EEA1 (*magenta*). Cells were visualized at ×63 magnification. The basal surface (*middle panel*) and midpoint of specified region (cross-section, *lower panel*) are presented to aid visualization of colocalization. Instances of colocalization between GLUT4 and TUSC5 without EEA1 are indicated by *closed arrowheads* and colocalization between all three proteins is indicated by *open arrowheads* (*top panel, scale bar,* 50 μm, *middle* and *bottom panel, scale bar,* 10 μm). *IP*, immunoprecipitation; *BAT*, brown adipose tissue; *Epi*, epididymal fat.

We used the well established GSV proteins GLUT4, IRAP (51), sortilin (10), and VAMP2 (52) to determine the characteristics of GSV proteins within our dataset. These proteins had three distinct features as follows: 1) they were present in all four replicates and were significantly enriched in GSVs under unstimulated conditions using the IgG control immunoisolation as a background value (enrichment) (Fig. 1*B*); 2) the abundance (LFQ intensity) of these proteins in GSVs from unstimulated adipocytes was above the median for the population (intensity) (Fig. 1*C*); and 3) their abundance was significantly reduced in GSVs isolated from insulin-stimulated adipocytes (insulin responsiveness) (Fig. 1*D*).

Using these criteria we identified 69 proteins that were enriched in GSVs (*n* = 4, integrated *p* < 0.05), 59 of which were above the intensity cutoff (mean log2 intensity >21.4933) (Fig. 1, *B*, *red* and *blue circles,* and *C*, *red* and *blue circles*, and supplemental Table S2). This list included proteins that regulate vesicle trafficking, including RAB proteins (1b, 5b, 11b, and 14), proteins involved in SNARE complex formation (syntaxins 6, 7, and 12 and VAMPs 2 and 3), and proteins involved in general trafficking processes (scamps 1 and 2, VTI1a, VPS26b, and VPS35) giving a cumulative profile of the GLUT4 trafficking itinerary. In view of the robustness of this filtering procedure, certain *bona fide* GSV proteins, including TBC1D4 (9), RAB10 (9, 16, 53), and LRP1 (16), were not included in this list, although they were identified in at least one of the four GSV preparations.

We measured reduced abundance of *bona fide* GSV residents in GSVs isolated from insulin-stimulated adipocytes due to the mobilization of GSVs to the plasma membrane. 23 of the 59 enriched proteins were insulin-responsive (*n* = 4, integrated, *p* < 0.05, see supplemental Table S3) (Fig. 1*D*). Notably, despite significant loss of GLUT4 from the immunoprecipitation starting material due to translocation of GLUT4 to the PM (observed in Fig. 2, *B* and *C*), not all coimmunoisolated proteins exhibited reduced abundance following insulin stimulation. The list of 23 insulin-responsive proteins included those previously found on GSVs such as syntaxin 6 (9, 16), syntaxin 7 (16), and syntaxin 12 (9, 16), RAB14 (9, 16), VTI1A (16), cystatin-3 (9, 16), and SCAMP2 (9, 16) and SCAMP1 (16) (Fig. 1*D*).

##### Validation of TUSC5 as a GSV Protein

Our analysis identified six novel GSV residents (supplemental Table S3). These were β_2_-microglobuli (B2M), α1-antiproteinase (SERPINA1), coatomer subunit α (COPA), granule cell differentiation protein/myotrophin (GDP/MTPN), apoptosis-inducing factor 2 (AIFM2), and TUSC5. *Tusc5* is a reported PPARγ target gene, and its expression is induced during adipocyte differentiation (54, 55), suggesting it may have a role in adipocyte function. Consistent with this, *Tusc5* mRNA is enriched in mouse and human adipose tissue (56). To confirm these findings, we performed immunoblotting with a TUSC5 antibody using a panel of mouse tissues. The antibody labeled one principal band of ∼20 kDa (Fig. 2*A,* *), similar to the predicted molecular mass of TUSC5 (18.7 kDa) as well as additional higher molecular mass bands of around 30 kDa (Fig. 2*A,* **). A similar molecular mass distribution was observed in 3T3-L1 adipocytes (Fig. 4*A*), and all bands were sensitive to siRNAs directed to TUSC5. These immunoreactive bands were only detected in adipose tissues with no detectable product in other tissues, including muscle and liver. Intriguingly, the higher molecular mass band was absent from BAT but abundant in epididymal fat.

TUSC5 levels in the LDM (Fig. 2*B*) or pHS (Fig. 2*C*) starting material was reduced in response to insulin, implying translocation of TUSC5 out of this subcellular compartment with insulin. TUSC5 was enriched in GSVs isolated from unstimulated adipocytes to a similar extent in both primary adipocytes and 3T3-L1 adipocytes, and the level of TUSC5 in GSVs was reduced with insulin stimulation similar to GLUT4 and IRAP. Notably, the single TUSC5-specific band identified in these immunoprecipitations was equivalent to the ∼20-kDa band in Fig. 2*A* (*).

We further corroborated TUSC5 localization to the GSV compartment by confocal microscopy in 3T3-L1 adipocytes (Fig. 2*D*). We analyzed unstimulated adipocytes because GSVs are highly insulin-responsive, and the number of GSVs is markedly reduced after stimulation. TUSC5 was present at the PM in unstimulated adipocytes as well as in the perinuclear region and in punctate cytosolic structures (Fig. 2*D*). There was considerable heterogeneity in the expression of TUSC5 between individual cells. The inter-cell variance in expression of GLUT4 and EEA1 was relatively low by comparison (Fig. 2*D, top panel*). There was a high level of colocalization between TUSC5 and GLUT4 in the perinuclear region and in smaller punctate peripheral structures in adipocytes (Fig. 2*D, middle* and *bottom panels*), but there was limited colocalization between TUSC5 and EEA1 (Fig. 2*D*, *open arrowheads*). Indeed, the majority of structures that were GLUT4- and TUSC5-positive were EEA1-negative (Fig. 2*D, middle* and *bottom panel, closed arrowheads*).

##### TUSC5 Translocates to the PM in Response to Insulin

**FIGURE 3.**
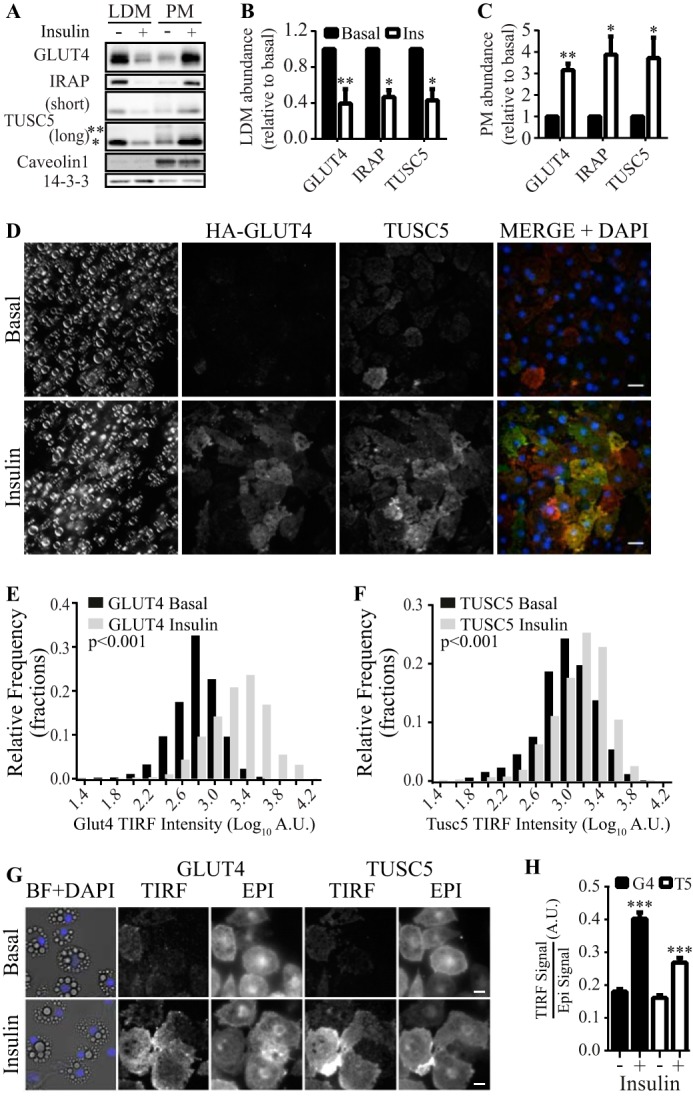
**TUSC5 translocates to the PM in response to insulin.** *A*, levels of GLUT4, IRAP, and TUSC5 were determined in purified LDM and PM subcellular fractions by immunoblotting. Subcellular fractions were generated from 3T3-L1 adipocytes treated with and without insulin as indicated. Two exposure levels are presented for TUSC5 to permit visualization of higher molecular mass forms of TUSC5 (* = ∼20 kDa, and ** = ∼30 kDa). To control for loading and to assess purity of the fractions, we immunoblotted for 14-3-3 and Caveolin1 in both fractions. *B*, quantification of GLUT4, IRAP, and TUSC5 levels in the LDM fraction in *A* relative to levels in unstimulated cells (*n* = 3, mean ± S.E., unpaired *t* test, *, *p* < 0.05; **, *p* < 0.01, all comparisons with basal values; *Ins*, insulin). *C*, quantification of GLUT4, IRAP, and TUSC5 levels in the PM fraction in *A* relative to levels in unstimulated cells (*n* = 3, mean ± S.E., unpaired *t* test, *, *p* < 0.05; **, *p* < 0.01, all comparisons with basal values). *Black* and *white bars* denote levels under basal and insulin conditions, respectively, as in *B. D*, 3T3-L1 adipocytes were serum-starved for 2 h and treated with 100 nm insulin for 20 min where indicated prior to fixation and processing for imaging by bright field light microscopy and immunofluorescent imaging by TIRF microscopy. Nonpermeabilized cells were stained for nuclei (DAPI), TUSC5, and HA-GLUT4 with anti-HA antibody. Cells were visualized at ×63 magnification (*scale bar,* 20 μm). *E*, histogram showing the distribution of TIRF signal intensity for HA-GLUT4 under basal conditions (*black bars*) and following insulin stimulation (*gray bars*) (Kolmogorov-Smirnov test comparing distributions under basal and insulin conditions, *p* < 0.001). *F*, histogram showing the distribution of TIRF signal intensity for TUSC5 under basal conditions (*black bars*) and following insulin stimulation (*gray bars*) (Kolmogorov-Smirnov test comparing distributions under basal and insulin conditions, *p* < 0.001). *G*, 3T3-L1 adipocytes were serum-starved for 2 h and treated with 100 nm insulin for 20 min where indicated prior to fixation and processing for imaging by bright field light microscopy (*BF*) and immunofluorescent imaging by epifluorescent (*EPI*) and TIRF microscopy (*TIRF*). Permeabilized cells were stained for nuclei (DAPI), TUSC5, and HA-GLUT4 with anti-HA antibody. Cells were visualized at ×63 magnification (*scale bar,* 20 μm). *H*, average TIRF signal/epifluorescent signal ratios for GLUT4 (*black bars*) and TUSC5 (*white bars*) under basal and insulin conditions as indicated (data from 158 to 295 cells from 10 regions of interest per condition from two independent experiments, mean ± S.E., Kolmogorov-Smirnov test, ***, *p* < 0.001). A.U., arbitrary units.

Because TUSC5 is an integral membrane protein localized to GSVs, we predicted that TUSC5 would undergo insulin-responsive translocation to the plasma membrane. In the absence of insulin, TUSC5 was enriched in the LDM fraction, and this was reduced by 57% in response to insulin. The reduction in TUSC5 levels in the LDM was similar to that observed for GLUT4 (−53%) and IRAP (−61%). There was a 3.7-fold increase in TUSC5 at the PM (Fig. 3, *A–C*) similar to that observed for other GSV cargo proteins (Fig. 3, *A–C*).

We detected a single ∼20-kDa TUSC5 band in the LDM(Fig. 3*A*, *), whereas multiple bands were detected in the PM (Fig. 3*A*, **). The increase in TUSC5 at the PM was dominated by the lower molecular mass of TUSC5 (Fig. 3*A*, *). The level of TUSC5 in the PM relative to the LDM in the absence of insulin was greater for TUSC5 than for either GLUT4 or IRAP (Fig. 3*A*). This was consistent with the distribution of TUSC5 observed by confocal microscopy (Fig. 2*D*).

We further interrogated TUSC5 translocation to the PM TIRF microscopy, which excites fluorophores in close proximity to the plasma membrane. By staining unpermeabilized adipocytes overexpressing the GLUT4 reporter construct HA-GLUT4 (50), we were able to specifically label PM TUSC5 and HA-GLUT4 levels in single cells (Fig. 3*D*).

To examine the change in abundance of HA-GLUT4 and TUSC5 at the PM in response to insulin, we plotted the distribution of TIRF signal intensities for HA-GLUT4 and TUSC5 from ∼800 cells under basal (*black bars*) and insulin-stimulated conditions (*gray bars*) (Fig. 3, *E* and *F*). Insulin stimulation significantly increased the number of cells with a higher TIRF signal intensity for HA-GLUT4. The same effect was observed for TUSC5. This indicated insulin-stimulated translocation of these proteins to the PM (Fig. 3, *E* and *F, gray bars*).

To further support this observation, we adapted the approach to quantify endogenous GLUT4 and TUSC5 translocation in permeabilized cells. This enabled assessment of the amount of GLUT4 and TUSC5 at the PM relative to their total expression levels (TIRF/epifluorescence) (Fig. 3*G*). Consistent with our other observations, insulin stimulation resulted in a 2.2- and 1.5-fold translocation of GLUT4 and TUSC5 to the cell periphery, respectively (Fig. 3*H*).

These data provide evidence that TUSC5 translocates from the LDM fraction to the PM in response to insulin. Furthermore, the magnitude of the TUSC5 and GLUT4 responses was similar, implying that they are delivered to the PM by the same carriers.

##### TUSC5 Is a Novel Positive Regulator of Insulin-stimulated Glucose Transport

**FIGURE 4.**
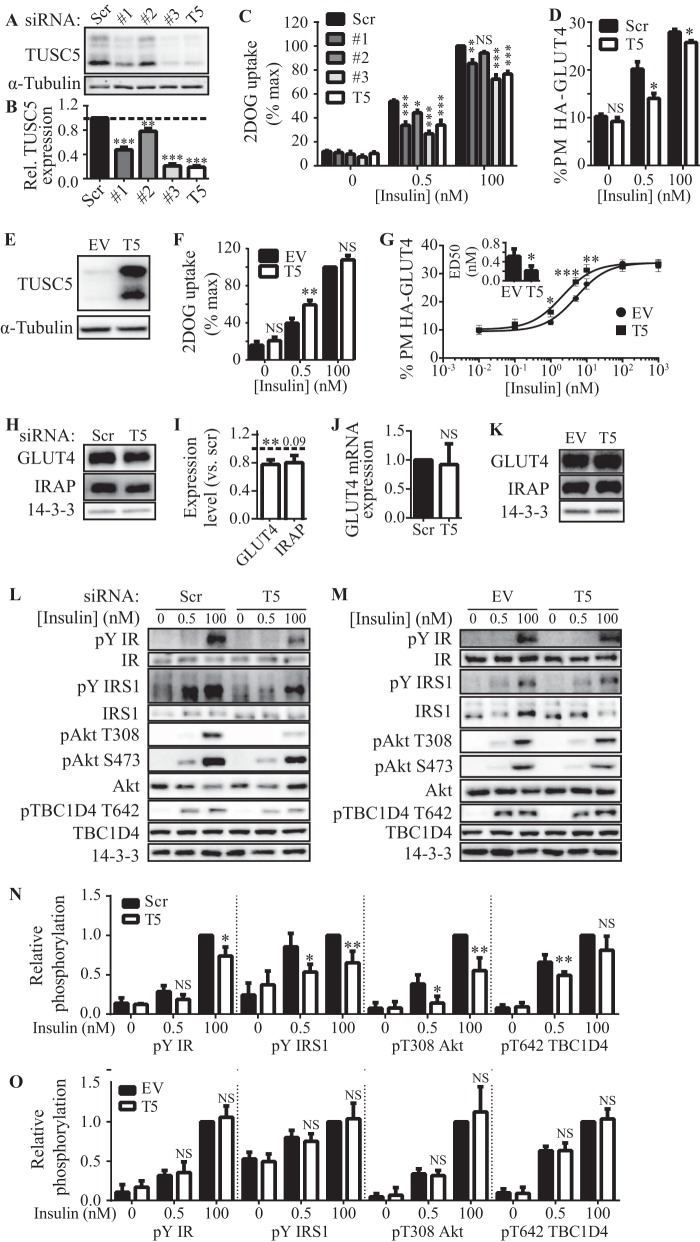
**TUSC5 is a positive regulator of insulin-stimulated GLUT4 trafficking.** *A*, total cell lysates from 3T3-L1 adipocytes treated with scrambled (*Scr*) or a panel of three different anti-*Tusc5* siRNAs (*#1, #2,* and *#3*), or a pool of all three siRNAs (*T5*) were immunoblotted for TUSC5 to determine the extent of TUSC5 knockdown. *B,* TUSC5 levels (in *A*) were quantified by densitometry and expressed as relative to cells treated with scrambled (*Scr*) siRNA (*n* = 4, mean ± S.E. one-way ANOVA; **, *p* < 0.01; ***, *p* < 0.001). *C*, 3T3-L1 adipocytes were treated with scrambled or individual anti-*Tusc5* siRNAs (*#1, #2,* and *#3*) or a pool of siRNAs (*T5*) for 96 h before 2DOG uptake assays were performed at the insulin doses indicated. 2DOG uptake expressed as a percentage of the maximum response in cells treated with scrambled siRNA (*n* = 4, mean ± S.E., two-way ANOVA; *NS,* nonsignificant; *, *p* < 0.05; **, *p* < 0.01; ***, *p* < 0.001, comparisons with control (*scr*) cells treated with the same insulin dose). *D*, PM levels of HA-GLUT4 in 3T3-L1 adipocytes overexpressing HA-GLUT4 treated with scrambled (*Scr*) or pooled *Tusc5* (*T5*) siRNA was measured using a fluorescence-based assay. Adipocytes were treated with insulin as indicated (*n* = 3, mean ± S.E., two-way ANOVA; *NS,* nonsignificant; *, *p* < 0.05, comparisons with control (*scr*) cells treated with the same insulin dose). *E*, retroviral overexpression of TUSC5 was assessed by immunoblotting whole cell lysates from cells expressing a control vector (empty vector, *EV*) or overexpressing TUSC5. α-Tubulin levels were determined as a loading control. *F*, 3T3-L1 adipocytes expressing a control vector (*EV*) or overexpressing TUSC5 were subjected to 2DOG uptake assays at the insulin doses indicated. 2DOG uptake expressed as a percentage of the maximum response in cells expressing control vector (*EV*) (*n* = 4, mean ± S.E., two-way ANOVA; *NS,* nonsignificant; **, *p* < 0.01, comparisons with cells expressing control vector (*EV*) treated with the same insulin dose). *G*, PM levels of HA-GLUT4 in 3T3-L1 adipocytes overexpressing HA-GLUT4 in combination with a control vector (*EV*) or TUSC5 were measured using a fluorescence-based assay. PM HA-GLUT4 levels in adipocyte-treated insulin doses as indicated were plotted as a dose-response curve (*n* = 3, mean ± S.E., two-way ANOVA; *NS,* nonsignificant; *, *p* < 0.05; **, *p* < 0.01; ***, *p* < 0.001 comparisons with cells expressing control vector (*EV*) treated with the same insulin dose). ED_50_ values for PM-GLUT4 were determined from nonlinear fitting of dose curves and graphed as an *inset* (*n* = 3, ± S.E., unpaired *t* test; *, *p* < 0.05, compared with cells expressing empty vector (*EV*)). *H*, GLUT4, IRAP, and 14-3-3 (loading control) levels were assessed by immunoblotting whole cell lysates from control (*scr*) and TUSC5 knockdown cells. *I*, GLUT4 and IRAP levels (*H*) were quantified by densitometry and are expressed as relative to control (*scr*) cells (*n* = 3, mean ± S.E., unpaired *t* test, **, *p* < 0.01, all comparisons with cells treated with scrambled siRNA; *dotted line* represents levels in cells treated with scrambled siRNA). *J*, mRNA expression of *Glut4* was determined by qPCR in cells treated with scrambled (*Scr*) and pooled anti-*Tusc5* (*T5*) siRNA. Data were expressed relative to levels in cells treated with scrambled siRNA (*n* = 3, mean ± S.E., unpaired *t* test; *NS,* nonsignificant, compared with cells treated with scrambled siRNA). *K*, levels of GLUT4, IRAP, and 14-3-3 (loading control) were assessed by immunoblotting whole cell lysates from control (*EV*) cells and cells overexpressing TUSC5. *L*, insulin signaling was monitored at the level of phosphorylation of insulin receptor (pIR), IRS1 (pIRS1), Thr(P)-308 and Ser(P)-473 AKT and Thr(P)-642 TBC1D4 in response to 0.5 and 100 nm insulin in control (*scr*) cells and cells treated with TUSC5 siRNA. Total levels of insulin receptor (*IR*), IRS1, AKT, and TBC1D4 were assessed in all conditions, and 14-3-3 was used as loading control. *M,* insulin signaling was monitored at pIR, pIRS, Thr(P)-308 and Ser(P)-473 AKT, and Thr(P)-642 TBC1D4 in response to 0.5 and 100 nm insulin in control (*EV*) cells and cells overexpressing TUSC5. Total levels of IR, IRS, AKT, and TBC1D4 were assessed in all conditions, and 14-3-3 was used as loading control. *N*, quantification of signaling intermediates in *L*. All data are expressed relative to substrate phosphorylation in response to 100 nm insulin in control (*scr*) cells (*n* = 3–5, mean ± S.E., unpaired *t* test; *NS,* nonsignificant; *, *p* < 0.05; **, *p* < 0.01, comparisons with cells expressing treated with scrambled siRNA under the same treatment conditions). *O*, quantification of signaling intermediates in *M*. All data are expressed relative to substrate phosphorylation in response to 100 nm insulin in control (*EV*) cells (*n* = 3, mean ± S.E., unpaired *t* test; *NS,* nonsignificant, comparisons with cells expressing treated with scrambled siRNA under the same treatment conditions).

Having confirmed TUSC5 localization to GSVs, we next investigated whether TUSC5 plays a role in insulin-regulated glucose transport. Because TUSC5 was similarly localized to GSVs in both primary and cultured adipocytes, we performed all subsequent analyses in 3T3-L1 adipocytes as these cells can be more readily manipulated to alter TUSC5 expression levels. We tested a panel of three siRNAs individually or in combination to assess their efficacy in reducing TUSC5 protein expression. All siRNAs reduced *Tusc5* expression levels (Fig. 4*A*), with siRNA #3 (−69%) and the pooled treatment (−71%) having greatest efficacy (Fig. 4*B*). To assess the role of TUSC5 in insulin action, we next examined insulin-dependent 2DOG uptake in siRNA-treated cells treated with an insulin dose equivalent to the ED_50_ value for glucose transport (0.5 nm) and a maximal dose (100 nm) to distinguish between effects on insulin sensitivity and the maximal response to insulin. In cells transfected with scrambled siRNA, insulin increased 2DOG uptake by 4.5- and 8.3-fold in response to 0.5 and 100 nm insulin, respectively (Fig. 4*C*). Knockdown of TUSC5 resulted in a significant impairment of insulin-stimulated 2DOG uptake. The degree of impairment in 2DOG uptake correlated with the level of knockdown as follows: siRNA #3 and the pooled siRNA treatment reduced insulin-stimulated 2DOG uptake by 50 and 38%, respectively, at 0.5 nm insulin and by 27 and 23%, respectively, at 100 nm insulin. For subsequent experiments, we used the combined siRNA treatment.

To explore this phenotype further, we examined insulin-regulated GLUT4 traffic (Fig. 4*D*) (50). In control cells, PM HA-GLUT4 was increased by 2.0- and 2.7-fold at 0.5 and 100 nm insulin, respectively. In TUSC5 knockdown cells, insulin-stimulated HA-GLUT4 translocation was significantly blunted at both 0.5 and 100 nm insulin.

The studies above indicated that loss of TUSC5 impairs insulin action in adipocytes. We next determined the effects of overexpression of TUSC5 on insulin action (Fig. 4*E*). TUSC5 overexpression markedly increased 2DOG uptake and HA-GLUT4 translocation at 0.5 nm insulin but not at 100 nm insulin (Fig. 4, *F* and *G*). A more detailed analysis of this effect revealed that TUSC5 overexpression enhanced insulin sensitivity as measured by a lowering of the ED_50_ value for insulin-stimulated GLUT4 translocation from 0.5 to 0.2 nm (Fig. 4*G, inset*).

To explore the mechanism for changes in 2DOG uptake in response to altered TUSC5 expression, we next examined GLUT4 expression levels in TUSC5 knockdown cells and in cells overexpressing TUSC5. GLUT4 protein levels were reduced by 21% in adipocytes treated with TUSC5-directed siRNA, although IRAP was not affected (Fig. 4, *H* and *I*). *Glut4* mRNA expression was unaffected in these cells (Fig. 4*J*). In contrast, we did not observe changes in GLUT4 or IRAP protein levels in response to TUSC5 overexpression (Fig. 4*K*).

We next examined how TUSC5 knockdown and TUSC5 overexpression affected insulin signaling responses. We monitored insulin signaling at the level of protein phosphorylation of proximal (insulin receptor, IRS1, and AKT) and more distal (TBC1D4) signaling elements by Western blotting using phosphosite-specific antibodies. Neither knockdown nor overexpression of TUSC5 resulted in a change in the total protein levels of any of the signaling molecules tested (Fig. 4, *L* and *M*). However, TUSC5 knockdown significantly inhibited insulin-stimulated phosphorylation of IRS1, AKT Thr-308, and TBC1D4 Thr-642 at 0.5 nm and of insulin receptor, IRS1, and AKT Thr-308 at 100 nm insulin (Fig. 4, *L* and *N*). In contrast, we detected no change in insulin signaling in cells overexpressing TUSC5 (Fig. 4, *M* and *O*), despite enhanced insulin sensitivity at the level of 2DOG uptake and HA-GLUT4 translocation (Fig. 4, *F* and *G*).

Loss of TUSC5 had pleiotropic effects on insulin-regulated glucose transport, thus inhibiting GLUT4 trafficking to the PM, reducing GLUT4 expression, and disrupting insulin signaling. However, the effect of TUSC5 overexpression was confined to enhancement of 2DOG uptake and GLUT4 translocation. Taken together, these genetic studies point toward TUSC5 playing a positive role in insulin-stimulated glucose transport at the level of regulated GLUT4 traffic. Inconsistencies in the effects on GLUT4 protein levels and insulin signaling responses in TUSC5 knockdown and overexpression models suggest that these factors are not responsible for changes in insulin sensitivity that occur with altered TUSC5 expression.

##### TUSC5 Is Required for the Insulin-sensitizing Effects of Rosiglitazone

**FIGURE 5.**
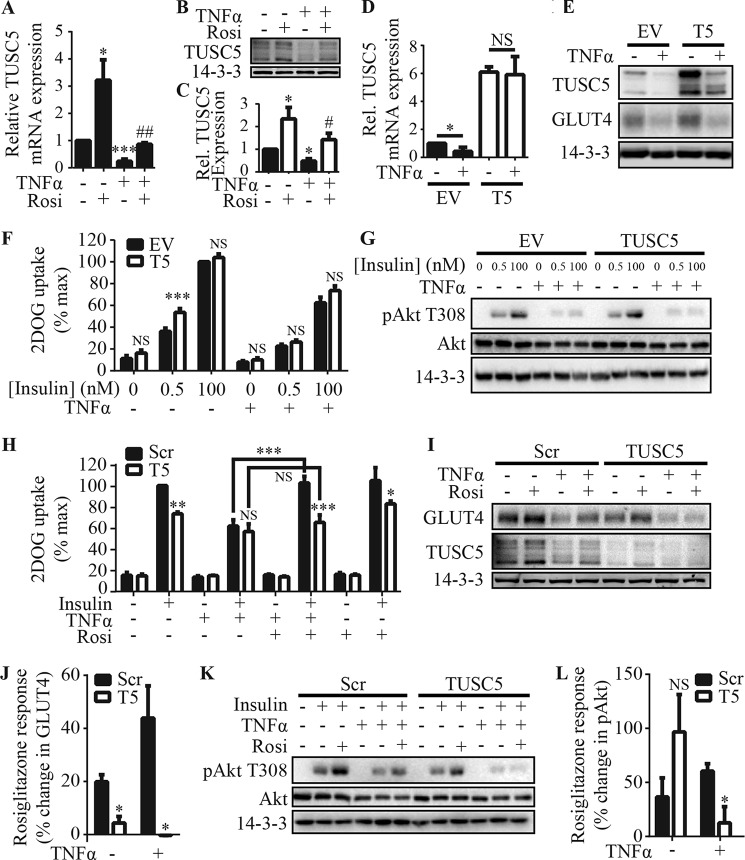
**TUSC5 is necessary, but not sufficient, for the insulin-sensitizing effects of rosiglitazone in insulin resistance.** *A*, *Tusc5* mRNA levels were determined by qPCR in cells treated with TNFα (2 ng/ml, 96 h) and/or rosiglitazone (10 μm, 48 h) as indicated. Data were expressed as relative to *Tusc5* levels in control cells (*n* = 3, mean ± S.E., unpaired *t* test; *, *p* < 0.05; ***, *p* < 0.001 for comparisons with untreated cells; ##, *p* < 0.05 for comparisons with TNFα-treated cells). *B*, TUSC5 levels were determined by immunoblotting in cells treated with TNFα (2 ng/ml, 96 h) and/or rosiglitazone (10 μm, 48 h) as indicated. α-Tubulin levels were determined as a loading control. *C*, TUSC5 abundance (*B*) was quantified by densitometry and expressed as relative to untreated cells (*n* = 4, mean ± S.E., unpaired *t* test; *, *p* < 0.05 for comparisons with untreated cells; #, *p* < 0.05 for comparisons with TNFα-treated cells). *D*, *Tusc5* mRNA levels were determined by qPCR in cells expressing an empty vector control (*EV*) or overexpressing TUSC5 (*TUSC5*) with and without TNFα treatment. Data were expressed relative to TUSC5 levels in untreated EV adipocytes (*n* = 3, mean ± S.E., unpaired *t* test; *NS,* nonsignificant; *, *p* < 0.05 for comparisons between untreated and TNFα-treated cells as indicated). *E*, TUSC5, GLUT4, and 14-3-3 (loading control) levels were assessed by immunoblotting whole cell lysates from cells expressing a control vector (*EV*) or overexpressing TUSC5 and treated with TNFα as indicated (representative of *n* = 4). *F*, 3T3-L1 adipocytes expressing a control vector (*EV*) or overexpressing TUSC5 were treated with TNFα (2 ng/ml, 96 h) as indicated before 2DOG uptake assays were performed. 2DOG uptake was expressed as a percentage of the maximum response (*n* = 4, mean ± S.E., two-way ANOVA; *NS,* nonsignificant; ***, *p* < 0.001; comparisons with cells expressing control vector (*EV*) under the same treatment conditions). *G*, 3T3-L1 adipocytes expressing a control vector (*EV*) or overexpressing TUSC5 were treated with TNFα (2 ng/ml, 96 h) as indicated before insulin signaling at the level of Thr(P)-308 AKT was monitored in response to 0.5 or 100 nm insulin (representative of *n* = 4). *H*, 3T3-L1 adipocytes were treated with scrambled or anti-*Tusc5* siRNA for 96 h and treated with TNFα (2 ng/ml, 96 h) and/or rosiglitazone (10 μm, 48 h) as indicated before 2DOG uptake assays were performed in unstimulated cells or cells stimulated with 100 nm insulin. 2DOG uptake was expressed as a percentage of the maximum response (*n* = 5, mean ± S.E., two-way ANOVA; *NS,* nonsignificant; *, *p* < 0.05; **, *p* < 0.01; ***, *p* < 0.001, comparisons with control (*scr*) cells under the same treatment conditions unless otherwise indicated). *I*, GLUT4, TUSC5, and 14-3-3 (loading control) levels were assessed by immunoblotting whole cell lysates from control (*scr*) and TUSC5 knockdown cells treated with TNFα and/or rosiglitazone as indicated. *J*, quantification of the change in GLUT4 expression in response to rosiglitazone in cells treated with scrambled (*Scr*) or pooled anti-*Tusc5* (*T5*) siRNA with and without TNFα treatment (*n* = 3, mean ± S.E., unpaired *t* test; *, *p* < 0.05, for comparisons with control (scr) cells under the same treatment conditions). *K,* 3T3-L1 adipocytes were treated with scrambled or anti-*Tusc5* siRNA for 96 h and treated with TNFα and/or rosiglitazone as indicated before insulin signaling in response to 100 nm insulin was monitored at the level of Thr(P)-308 AKT (representative of *n* = 3). *L,* quantification of the change in insulin-stimulated phosphorylation of AKT at Thr-308 in response to rosiglitazone in cells treated with scrambled (*Scr*) or pooled anti-*Tusc5* (*T5*) siRNA with and without TNFα treatment (*n* = 3, mean ± S.E., unpaired *t* test; *NS,* nonsignificant; *, *p* < 0.05 for comparisons with control (*scr*) cells under the same treatment conditions).

*Tusc5* expression levels are decreased in epididymal white adipose tissue of obese rats (56). Furthermore, it has been reported that *Tusc5* expression is regulated by PPARγ (54, 55). PPARγ agonists, such as the thiazolidinediones (*e.g.* rosiglitazone), can reverse insulin resistance in adipocytes (20–24). However the mechanisms by which PPARγ activation confers this benefit are not fully understood (20–32). In light of these data and our finding that TUSC5 regulates insulin-stimulated glucose transport in adipocytes (Fig. 4), we hypothesized that TUSC5 expression may be reduced in insulin resistance and that PPARγ agonists may work in part by restoring TUSC5 expression levels. To study this, we used the TNFα model of insulin resistance in 3T3-L1 adipocytes, which has been reported to be reversed by treatment with the PPARγ agonist pioglitazone (21). TNFα markedly reduced *Tusc5* mRNA (−76%) and protein levels (−54%), and *Tusc5* expression was rescued at both levels by cotreatment with rosiglitazone (Fig. 5, *A–C*).

We assessed whether rescue of TUSC5 expression was sufficient to overcome TNFα-induced insulin resistance. Overexpression of *Tusc5* under the control of an exogenous promoter rendered *Tusc5* mRNA insensitive to TNFα (Fig. 5*D*). We observed a small TNFα-induced reduction in TUSC5 protein expression (Fig. 5*E*), but TUSC5 expression was maintained at levels in untreated control cells (Fig. 5*E*). Notably, different molecular weight forms of TUSC5 appeared differentially sensitive to TNFα (Fig. 5*E*). Rescue of TUSC5 expression did not protect adipocytes from TNFα-induced insulin resistance (Fig. 5*F*), loss of GLUT4 (Fig. 5*E*), or inhibition of insulin signaling (Fig. 5*G*).

We next determined whether TUSC5 was necessary for the reversal of TNFα-induced insulin resistance by the PPARγ agonist rosiglitazone. In untreated cells, rosiglitazone did not enhance insulin-stimulated 2DOG uptake (Fig. 5*H*) despite increasing GLUT4 expression (+20%) (Fig. 5, *I* and *J*) and insulin signaling (+37%) (Fig. 5, *K* and *L*). TNFα treatment caused insulin resistance as indicated by a 44% reduction in insulin-stimulated 2DOG uptake (Fig. 5*H*), loss of GLUT4 (−48%, Fig. 5*I*), and reduced insulin-regulated AKT phosphorylation (−54%, Fig. 5*K*). Rosiglitazone reversed TNFα-induced defects in insulin-stimulated 2DOG uptake in adipocytes that were transfected with scrambled siRNA (Fig. 5*H*). Furthermore, rosiglitazone increased GLUT4 expression by 44% (Fig. 5, *I* and *J*) and insulin-mediated AKT phosphorylation at Thr-308 by 60% (Fig. 5, *K* and *L*) in these cells.

These data provided a response profile from which we could determine whether loss of TUSC5 altered adipocyte responses to rosiglitazone. In untreated cells, TUSC5 knockdown blocked GLUT4 expression in response to rosiglitazone (Fig. 5*J*) but did not prevent enhanced in signaling to AKT (Fig. 5, *K* and *L*). In contrast, TUSC5 knockdown completely blocked all rosiglitazone responses in TNFα-treated cells so that no rosiglitazone response was measured at the level of insulin-stimulated 2DOG uptake (Fig. 5*H*), GLUT4 expression (Fig. 5, *I* and *J*), or insulin signaling (Fig. 5, *K* and *L*).

## Discussion

We performed proteomic analysis of the GLUT4 storage compartment to identify novel GSV cargo and regulators of insulin-stimulated glucose transport. These studies led to the identification of six proteins not previously reported to be GSV residents. One of these proteins, TUSC5, was highly enriched in adipose tissue, underwent insulin-dependent translocation to the PM-like GLUT4, and was found to be a novel regulator of insulin-regulated glucose transport. Furthermore, *Tusc5* is a PPARγ target gene, and we provide evidence that TUSC5 is required for PPARγ agonism to overcome adipocyte insulin resistance.

Our analysis of the GSV proteome yielded 23 proteins that we have designated as high confidence GSV proteins (supplemental Table S3). Although there have been previous proteomic analyses of the adipocyte GSV proteome (9, 16), our analysis is the first to take into account the “intensity” and “insulin responsiveness” of proteins to increase confidence of hits being part of the GSV compartment. The filtering process enabled us to identify proteins that exhibit similar features to *bona fide* GSV proteins, such as GLUT4, and as such are biased toward proteins that are enriched in basal GSVs to the exclusion of proteins that may, for example, specifically interact with GSVs following insulin stimulation.

Although the majority of the 23 GSV proteins reported here have been described previously (supplemental Table S3) (9, 16), we identified six novel GSV proteins. B2M is a component of the class I MHC complex (57, 58), and SERPINA1 is a proteinase inhibitor (59), and both are secreted proteins. The class I MHC has been reported to translocate to the PM in response to insulin in adipocytes, although it did not colocalize with GLUT4 under basal conditions (60). COPA is a cytosolic protein involved in the budding of vesicles from the Golgi (61), and GCDN/MTPN binds to actin capping protein (62) and has been reported to be involved in neurotransmitter release in neurons and glucose-stimulated insulin secretion in beta cells (63, 64). AIFM2 is a single-pass transmembrane domain protein. AIFM2 has oxidoreductase activity (65) and has been reported to be a P53 target gene (66) and mediator of caspase-independent apoptosis (67).

TUSC5 is dual-pass membrane protein and was of particular interest as it was reported to be highly expressed in adipose depots and to be a target of PPARγ, a master regulator of adipocyte function (54–56). It was initially described to be a deleted gene in lung cancer (68), although there is little known about its function. It possesses a large extracellular domain that contains one single *N*-linked glycosylation site. TUSC5 also possesses a C-terminal di-Lys motif. These motifs often encode binding sites for the coatomer coat complex, which is of interest because one of the subunits of this complex, COPA, was identified as a GSV component. Although coatomer is traditionally thought of as a regulator of endoplasmic reticulum to Golgi vesicle transport, there is also evidence that it plays a role in endosomes (69). Hence, it will be of interest to explore the role of coatomer in GLUT4 trafficking in fat cells.

TUSC5 was validated as a GSV protein in primary and 3T3-L1 adipocytes by immunoprecipitation, and we observed a high degree of TUSC5 colocalization with GLUT4 in a nonendosomal compartment (EEA1-negative) in 3T3-L1 adipocytes. Furthermore, TUSC5 translocated from the LDM fraction to the PM and also did to a similar extent to GLUT4. These data further support its localization to the same carrier as GLUT4.

One key difference between the subcellular localization of TUSC5 and GLUT4 was the relatively large degree of PM TUSC5 under basal conditions. This raises the question of where TUSC5 elicits its effect on insulin action. Its colocalization with GLUT4 in GSVs and its potential for interacting with coatomer potentially places its action within the cell. That loss of TUSC5 resulted in reduced GLUT4 protein levels, independent of *Glut4* mRNA expression, and impaired GLUT4 trafficking in TUSC5 knockdown cells might indicate a role for TUSC5 in GSV biogenesis. Loss of other integral GSV proteins, IRAP and LRP1, also result in loss of GLUT4 proteins (16, 70). Alternatively, TUSC5 may also play a role at the PM. Intriguingly, a portion of TUSC5 found on the PM appeared to have a higher molecular weight than that found in GSVs (Fig. 3*A*), raising the possibility that it undergoes post-translational modification either prior to or in response to movement to the cell surface.

Several pieces of evidence were obtained here to implicate TUSC5 as a positive regulator of insulin action in adipocytes. Knockdown of TUSC5 using siRNA resulted in reduced sensitivity to insulin, although TUSC5 overexpression had the opposite effect, halving the ED_50_ value for GLUT4 translocation. Although knockdown of TUSC5 resulted in a complex array of additional changes, including reduced GLUT4 expression and impaired insulin signaling, overexpression did not affect these parameters. Therefore, we propose that the effect of TUSC5 expression on insulin-regulated GLUT4 traffic is likely not mediated by changes in GLUT4 expression or signaling. Rather observed changes in response to TUSC5 knockdown may be downstream of the defects in GLUT4 trafficking. How TUSC5 acts as an insulin sensitizer is the subject of ongoing work. Our genetic experiments cannot distinguish between an acute and chronic role for TUSC5 in modulating insulin action. However, the breadth of responses to TUSC5 knockdown (Fig. 4) and because TUSC5 is required for PPARγ-mediated reversal of insulin resistance (Fig. 5) suggest a longer term role in modulating insulin responses in adipocytes. Of interest, we observed considerable heterogeneity in TUSC5 expression between individual adipocytes (Fig. 2) and as to whether this is linked to the heterogeneity in insulin action that has been described in 3T3-L1 adipocytes (71) remains to be seen.

PPARγ plays an important role as an insulin sensitizer in adipocytes (17–19), although the exact mechanism by which this is achieved or which PPARγ target genes confer this benefit has not been fully resolved. We have recently shown that insulin resistance in adipocytes is highly selective for GLUT4 trafficking, and so the ability of PPARγ agonists to reverse insulin resistance would seem to reflect an important role for PPARγ in regulating components of the GLUT4 trafficking process (72). We have shown that TUSC5 expression was reduced in insulin resistance, and although TUSC5 was not sufficient to overcome insulin resistance, TUSC5 was necessary for the full insulin-sensitizing effects of rosiglitazone. Our findings that TUSC5 is highly regulated by PPARγ, is expressed at such high levels in adipose tissue, and that its expression is required for PPARγ-mediated reversal of insulin resistance emphasizes the essential role of this protein in adipocyte function and in particular the effects of PPARγ agonists in this tissue. Furthermore, it is conceivable that reduced levels of TUSC5 may contribute to the development of insulin resistance.

However, there are several lines of evidence that suggest that enhanced TUSC5 expression is not the only effect of PPARγ agonism. First, overexpression of TUSC5 did not recapitulate the effects of rosiglitazone. Second, TUSC5 expression was not required for rosiglitazone to enhance insulin signaling in control cells (Fig. 5, *K* and *L*) despite considerable up-regulation of TUSC5 in response to rosiglitazone (Fig. 5*A*). It is known that rosiglitazone can influence signaling via additional mechanisms such as through the induction of a number of signaling intermediates (23). Therefore, the requirement for TUSC5 for the insulin-sensitizing effects of rosiglitazone appears to be context-dependent.

Overall, we provide evidence that TUSC5 is a novel GSV protein and regulator of insulin-stimulated glucose transport responses in adipocytes. TUSC5 is the first PPARγ target gene, other than GLUT4 itself, to provide a direct link between PPARγ activation and the regulation of glucose transport. Understanding the mechanism by which TUSC5 interacts and regulates with insulin-regulated GLUT4 traffic may provide novel insight into this system and the defects associated with insulin resistance.

## Author Contributions

D. J. F., F. K., M. J. P., G. D. H., and D. E. J. conceived of the study. D. J. F., S. N., F. K., and B. L. P. performed proteomic experiments for Fig. 1. S. N. and R. C. analyzed proteomic data for Fig. 1. D. J. F., S. N., F. K., and J. G. B. designed, performed, and analyzed the experiments shown in Fig. 2. J. S., C. C. M., and B. A. M. performed experiments for Fig. 2. D. J. F., S. N., and J. G. B. designed, performed, and analyzed the experiments shown in Fig. 3. D. J. F., S. N., J. R. K., and K. C. T. designed, performed, and analyzed the experiments shown in Fig. 4. D. J. F., S. N., and K. C. T. designed, performed, and analyzed the experiments shown in Fig. 5. D. J. F., S. N., and D. E. J. wrote the paper. All authors reviewed the results, edited the manuscript, and approved the final version of the manuscript.

## Footnotes

6 The abbreviations used are:

PM: plasma membrane
EEA1: early endosomal antigen 1
GLUT4: glucose transporter type 4
GSV: GLUT4 storage vesicle
HDM: high density microsome
IRAP: insulin-responsive aminopeptidase
LDM: low density microsome
LFQ: label-free quantification
pHS: post-HDM supernatant
PPAR: peroxisome proliferator-activated receptor
TEAB: triethylammonium bicarbonate
2DOG: 2-[^3^H]deoxyglucose
ANOVA: analysis of variance
TIRF: total internal reflection fluorescence
qPCR: quantitative PCR.
